# Cancer-associated fibroblasts in radiotherapy: challenges and new opportunities

**DOI:** 10.1186/s12964-019-0362-2

**Published:** 2019-05-17

**Authors:** Zhanhuai Wang, Yang Tang, Yinuo Tan, Qichun Wei, Wei Yu

**Affiliations:** 1 Department of Surgical Oncology, Second Affiliated Hospital, Zhejiang University School of Medicine, Hangzhou, Zhejiang 310009 China; 2 Cancer Institute (Key Laboratory of Cancer Prevention and Intervention, China National Ministry of Education), The Second Affiliated Hospital, Zhejiang University School of Medicine, Hangzhou, Zhejiang 310009 China; 3 Department of Radiation Oncology, Second Affiliated Hospital, Zhejiang University School of Medicine, Hangzhou, 310009 Zhejiang China

**Keywords:** Tumor microenvironment, Cancer-associated fibroblasts, Radiotherapy

## Abstract

**Background:**

Radiotherapy is one of the most important therapeutic strategies for treating cancer. For decades, studies concerning the outcomes of radiotherapy mainly focused on the biological effects of radiation on tumor cells. Recently, we have increasingly recognized that the complex cellular interactions within the tumor microenvironment (TME) are closely related to treatment outcomes.

**Main content:**

As a critical component of the TME, fibroblasts participate in all stages of cancer progression. Fibroblasts are able to tolerate harsh extracellular environments, which are usually fatal to all other cells. They play pivotal roles in determining the treatment response to chemoradiotherapy. Radiotherapy activates the TME networks by inducing cycling hypoxia, modulating immune reaction, and promoting vascular regeneration, inflammation and fibrosis. While a number of studies claim that radiotherapy affects fibroblasts negatively through growth arrest and cell senescence, others argue that exposure to radiation can induce an activated phenotype in fibroblasts. These cells take an active part in constructing the tumor microenvironment by secreting cytokines and degradative enzymes. Current strategies that aim to inhibit activated fibroblasts mainly focus on four aspects: elimination, normalization, paracrine signaling blockade and extracellular matrix inhibition. This review will describe the direct cellular effects of radiotherapy on fibroblasts and the underlying genetic changes. We will also discuss the impact of fibroblasts on cancer cells during radiotherapy and the potential value of targeting fibroblasts to enhance the clinical outcome of radiotherapy.

**Conclusion:**

This review provides good preliminary data to elucidate the biological roles of CAFs in radiotherapy and the clinical value of targeting CAFs as a supplementary treatment to conventional radiotherapy. Further studies to validate this strategy in more physiological models may be required before clinical trial.

## Background

Radiotherapy is one of the most important therapeutic strategies used for the treatment of more than two-thirds of cancer cases in western countries. In fact, radiotherapy is the curative treatment regimen for some early stage tumors (e.g. non–small-cell lung cancer) [1]. Over the past few decades, advances in technology propelled the development of 3-dimentional conformal radiation techniques including intensity-modulated radiation therapy (IMRT) and stereotactic body radiation therapy (SBRT). Today, the combination of 3-dimentional conformal radiation techniques and advanced imaging systems allows for matched radiation doses to be accurately delivered to precise positions of the lesion; sparing adjacent normal tissue to the maximum extent [2]. In addition to these cutting-edge technologies, our knowledge of tumor biology and radiobiology has grown significantly with advances in immunology, physiology and molecular cell biology, allowing us to analyze the treatment outcome from a more comprehensive perspective and perform radiotherapy more efficiently. In the past two decades, radiotherapy has greatly contributed to the improvement of overall survival in malignant tumors such as head and neck squamous cancer [2, 3]. However, primary resistance and acquired resistance shortly after treatment remain a great challenge and must be addressed to further improve radiotherapy efficacy.

Early studies in radiobiology mainly focused on tumor cells, overlooking the complex cellular interactions that occur within the tumor microenvironments (TME) [4]. Cancer associated fibroblasts refers to a heterogeneous group of stromal cells in the tumor which are phenotypically and epigenetically different from normal fibroblasts. While the term “fibroblast” refers to a specific type of cell, cancer associated fibroblasts are in fact morphologically fibroblast-like cells that origin from different tissues or precursor cells [5–7]. The major source of CAFs are normal fibroblasts, which are transformed by the tumor microenvironment. In addition, stellate cells, bone-marrow-derived fibrocytes, mesenchymal stem cells, and even endothelial cells, adipocytes, pericytes and smooth muscle cells have been reported to be cellular origins of CAFs [5, 7]. The histological identity of CAFs are generally based on positive staining of molecular markers such as α-SMA, FAP, S100A4 and platelet derived growth factor receptor-β (PDGFR-β). However, many of these markers are present in normal activated fibroblasts and other cells types, and do not serve as exclusive markers for CAFs [5, 7, 8]. CAFs exert their biological functions by modulating the extracellular matrix and by secreting cytokines or growth factors that regulate tumor proliferation, invasion and metastasis. While early studies claim that CAFs are pro-tumorigenic, increasing evidence suggests that some CAFs may inhibit tumor progression, possibly by forming a physical barrier to restrict tumor cell growth and migration [9]. The heterogeneity of CAFs has recently attracted much attention, and efforts have been made to discriminate and classify different CAFs subpopulations in various types of cancers. Four types of CAFs have been verified in breast cancer based on the expression of Integrinβ1, α-SMA, FSP1, FAP, PDGFR and Caveolin1 [10]. Five CAFs subtypes have been described in lung cancer using RNA sequencing, each featuring a specific expression pattern of collagen types with distinct expression of genes related to myogenesis [11]. In pancreatic cancer, CAFs have been divided into α-SMA expressing (myCAFs) and IL6 expressing (iCAFs) CAFs [12]. Whether the subgroups of CAFs described in these studies are the result of lineage differentiation or merely different phenotypes induced by a specific set of stimuli remains unclear. Given the diversity of CAFs, it would be intriguing to further define each subgroup by more comprehensive means such as single-cell based genomics and proteomics [8].

Radiotherapy leads to a series of changes in the TME networks including cycling hypoxia, immune modulation, vascular regeneration, inflammation and fibrosis [4]. While a number of studies claim that radiotherapy impacts fibroblast negatively through growth arrest and cell senescence, others suggest that radiotherapy promotes activation of normal fibroblasts through inducing a senescence-like phenotype. Radiotherapy promotes tumor stromal fibroblasts activation by inducing DNA damage, generating reactive oxygen species and initiating inflammation cascades [13]**.** Radiation induced senescence-like fibroblasts differ from replicative senescence cells in that no telomere shortening is detected [14]. Nevertheless, they share similar characteristics with activated fibroblasts or can be termed more directly as CAFs-like cells.

Therapeutic resistance in cancer is defined as a process in which cancer lesions progress concurrent with or secondary to an initial response to therapeutic intervention. It is the major reason for poor prognoses of cancer. Interactions between tumor cells and stromal cells are considered to be an important cause of radiation- and chemotherapy resistance. While traditional radiobiology generally overlooks the considerable effect of radiation treatments on the TME as well as the various cytokines that the TME release as a feedback. [2] Evidences from pre-clinical studies have indicated that radiation induced changes in the TME favor tumor progression in certain tumor models. The murine mammary epithelial cell line, COMMA-D, which are non-tumorigenic, could form visible large tumors when injected into radiation pre-treated fat pads in mice [15]. Thus, tumor microenvironments may also contribute to tumorgenesis and play critical roles in determining the outcomes of radiotherapy [16]. Recent studies have shown that secreted factors produced by CAFs induce chemotherapy resistance in tumor cells [17–20]. Given the direct and indirect role that CAFs play in therapeutic resistance, it is essential to explore the role of fibroblasts in radiotherapy resistance [21]. This review will describe the direct cellular effects of radiotherapy on CAFs and the underlying genetic changes. We will also discuss the effect of CAFs on cancer cells during radiotherapy and the potential value of targeting CAFs to enhance the clinical outcome of radiotherapy. Main findings of the role that CAFs play in radiotherapy are summarized in Table 1.

### Radiotherapy causes cytostatic but not cytolytic effects on CAFs

**Fig. 1:**
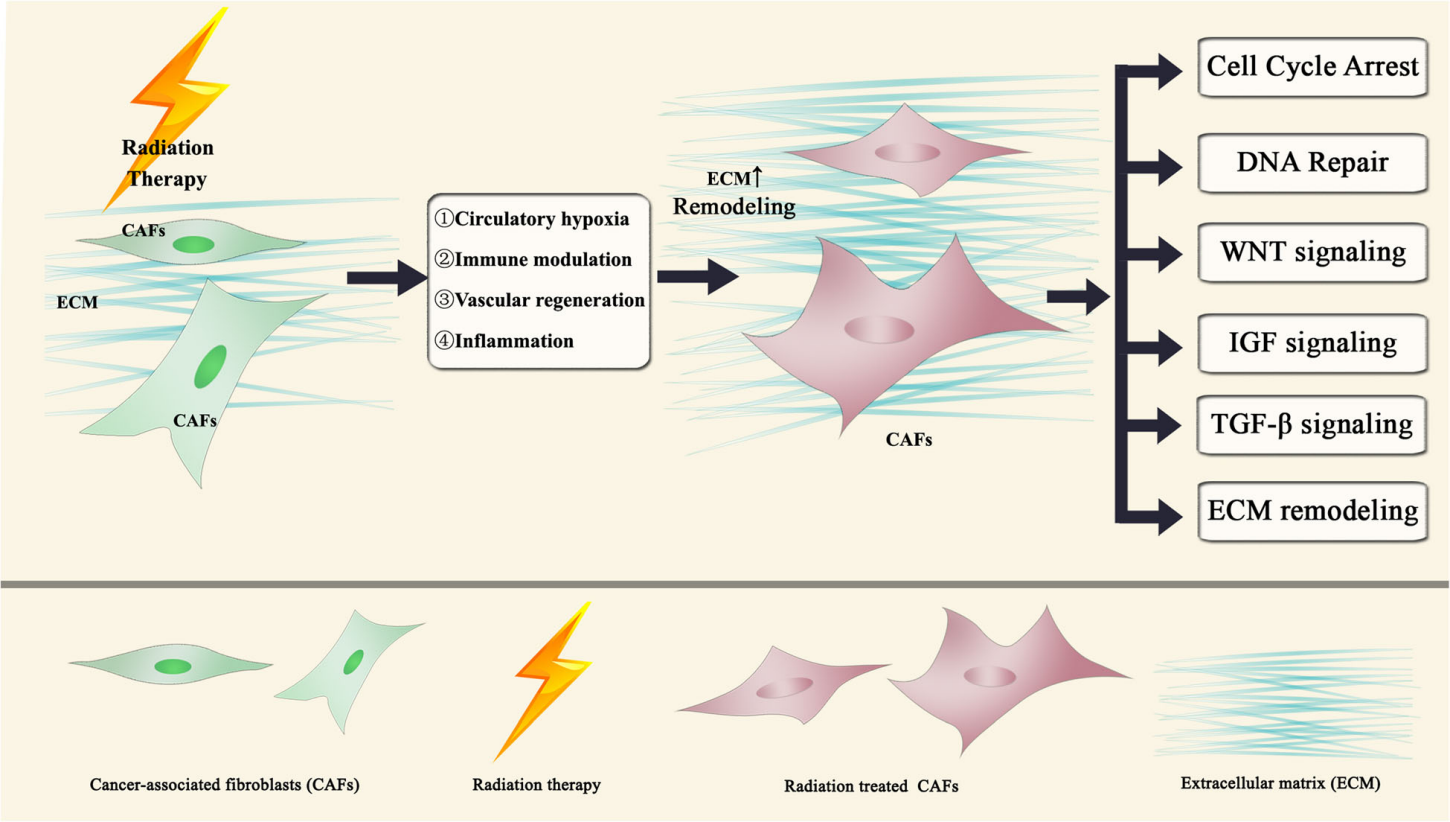
Radiation causes a series activation process in tumor microenvironment (TME) including cycling hypoxia, immune modulation, vascular regeneration, inflammation and fibrosis. Radiation therapy induces fibroblast into senescence-like fibroblasts, which share similar characteristics with activated fibroblasts. These cells work together to remodeling the TME. High levels of TGF-β1 was detected in conditioned media from irradiated fibroblasts, which promoted their self-activation. The altering gene expression in radiation treated fibroblasts are mainly focus on cell cycle arrest, DNA repair, ROS scavenging, ECM remodeling, Wnt signaling and IGF signaling

Studies investigating the direct cytotoxic effects of fractionated radiotherapy on CAFs have found that fibroblasts are naturally resistant to radiation. Tommelein et al. treated primary CAFs from colorectal cancer with scheduled (fractionated dose 5 × 1.8Gy or 10 × 1.8Gy) radiotherapy which induced DNA damage, p53 activation, cell-cycle arrest in the CAFs. However, none of the regimens caused obvious cell death or changes in morphological appearances. The α–SMA expression and the CAFs’ ability to contract collagen gel was also not affected by any of the radiation regimens [22]. In stereotactic ablative radiotherapy (SART), intensive doses of radiation are used to ablate cancer cells. Hellevik et al. compared the radiotherapy sensitivity of CAFs and (non-small cell lung cancer) NSCLC by using single dose radiation (2, 6, 12 or 18 Gy) or fractionated radiation regimens (6 × 3 Gy). Results indicated that none of the above radiation regimens caused cell death within 3 weeks of treatment. Anti-53BP-1 staining showed that radiation caused DNA damage in CAFs in a dose dependent manner (18Gy > 12Gy > 6 Gy > 2Gy). Moreover, a single dose of radiation at 18 Gy (ablative doses) caused persistent DNA damage compared with the 6 × 3 Gy. And β-galactosidase staining showed that the single dose treatment caused a more pronounced senescence response in the CAFs than the fractionated treatment [23] (Fig. 1). These results suggest that radiotherapy does affect the proliferation of CAFs at the genetic level but leaves the CAFs viable to sustain an active tumor microenvironment which may support the growth of resilient tumor cells.

**Table 1:** Main findings of the cross talk between CAFs and cancer cells during radiotherapy

Author	Ref.	Fibroblasts source	Main findings
Tommelein et al.,2017	[22]	CRC	1. RT cause DNA damage, p53 activation, cell-cycle arrest and IGF-1 secretion in CAFs 2. RT-activated CAF promoted CRC cells survival and metabolic switch
			3. RT followed by IGF-1R neutralization reduced organ metastases in orthotropic model
			4. mTOR was significantly higher in Rectal Cancer patients after neoadjuvant CRT
Zhang et al., 2017	[37]	ESCC and adjacent normal tissue	1. CAFs induced radioresistance by secreting CXCL1
			2. CXCL1 expression was an independent prognostic factor for patients receiving CRT
Grinde et al., 2017	[26]	NSCLC	1. CAFs viability, adhesion did not affected by single-high dose radiation and fractionated doses radiation.
			2. Radiation abolished CAFs protumorigenic capacity in the coinjection model in mice.
Wang et al., 2017	[38]	Foreskin, lung cancer	CAF promoted tumor cells DNA damage repair after radiation via mTOR-mediated autophagy
Li et al., 2016	[31]	Pancreatic cancer and adjacent normal tissue	1. Radiation enhanced CAFs migration- and invasion-promoting capacity
			2. CXCL12 secreted by CAFs was increased after radiation, which enhanced tumor invasion and EMT
Verset et al., 2015	[34]	Rectal cancer	1. Patients underwent neoadjuvant CRT showed higher a-SMA/neoplastic epithelial area ratio
			2. The alpha-SMA/epithelial area ratio above 1 is associated with poor recurrence-free survival
(Matsuoka et al., 2015	[33]	OSCC	High level of CAFs were correlated with advanced pT- and pN- stage and poor prognosis
Ji et al., 2015	[51]	NSCLC	1.CAFs promoted cocultured lung cancer cells radioresistance in vitro
Bao et al., 2015	[32]	ESCC and adjacent normal tissue	2.Irradiated fibroblasts increased ESCC cells invasiveness with decreased E-cadherin and increased vimentin expression
Arshad et al. 2015	[28]	primary murine lung fibroblasts	CM from irradiated fibroblasts did not change TC-1 cell radiation sensitivity but stimulated their migration and increased their Vimentin and Snail expression
Chu et al. 2014	[36]	CESE	1.CM from CAFs increased irradiated Hela cell survival
			2.Such effect was enhanced by using CM from mixed culture of CAFs and HeLa cells
Boelens et al., 2014	[41]	MRC-5 fibroblasts	1.Exosomes transferred from MRC-5 fibroblasts induced the interferon-related DNA damage resistance signature (IRDS) in breast cancer cells
			2. In xenograft mouse model, coinjected MRC-5 fibroblasts protected breast cancer cells from 8 Gy RT and maintained tumor growth compared to single breast cancer cell group.
Hellevik et al., 2013	[25]	NSCLC	1. Ablative ionizing radiation (AIR) on CAFs result in
			1) downregulated secretion of angiogenic factors such as SDF-1, angiopoietin, and TSP-2
			2) upregulated secretion of bFGF;
			3) unaffected expression levels of HGF, IL-1β, IL-6, IL-8 and TNF-α
			2.Irradiated CAFs did not affect H-520/H-522 proliferation or migration
			3. CM from irradiated CAFs reduced HUVECs cells migration
Hellevik et al., 2012	[23]	NSCLC	1. AIR induced cellular senescence and inhibited proliferation, migration and invasion in CAFs.
			2.AIR promoted MMP-3 and inhibited MMP-1 expression in CAFs
			3. AIR enhanced CAFs surface expression of integrin 2, 1 and 5
Saigusa et al., 2011	[35]	Rectal cancer	1. In rectal cancer, FAP-α and SDF-1 were mainly expressed in CAFs
			2. Positive expression of FAP-α and SDF-1 predicted distant recurrence
Kamochi et al., 2008	[29]	*Wi26 VA4* fibroblast (SV40-transformed	1. Radiation reduced both the number of NIH 3 T3 and WI-26 VA4 fibroblasts after 15 days.
		human lung fibroblasts); NIH 3T3 fibroblast	2. Irradiated fibroblasts enhanced the invasion of SCC cells and had no impact on their apoptosis
			3. Irradiated fibroblasts expressed high TGF-1 compared to control
Weichselbaum et al., 2008	[40]	MRC-5 fibroblast	Exosomes transferred from MRC-5 fibroblast activated IRDS and anti-viral/NOTCH3 pathway in breast cancer cells to enhance their radioresistance in vitro and in mice models
Ohuchida et al., 2004	[30]	human fibroblast cell line MRC5, primary pancreatic fibroblasts	1. 5-Gy/10-Gy irradiation on fibroblasts partially inhibited proliferation but caused no cytolytic after 24 h
			2. Irradiated fibroblasts enhanced pancreatic cancer cells invasiveness in a radiation dose(0, 5 10 Gy) dependent manner
			3. Irradiated fibroblasts increased both phosphorylation and expression of c-Met as well as the MAPK activity of pancreatic cancer cells

### Radiotherapy influences gene expression in fibroblasts

Data concerning gene expression changes of CAFs after radiotherapy are limited. Nevertheless, altered gene expression profiles of irradiated normal fibroblasts has been explored in a number of early studies. Rodningen et al. evaluated gene expression changes in irradiated normal fibroblasts derived from skin biopsies from 30 different individuals. Fibroblasts were treated with either single dose 3.5 Gy or 10.5 Gy radiation in 3 consecutive daily fractions. Pathways that responded to radiation were associated with the following biological processes: (1) Cell cycle arrest, mainly induced by ATM and p53; (2) DNA repair, a critical process after ionizing radiation which repeated itself inside the cell; (3) ROS (reactive oxygen species) scavenging, which participates in radiation induced biological processes; (4) ECM remodeling, a process closely related to radiation induced fibrosis and fibroblast differentiation; (5) Wnt signaling, which partially regulates radiation induced matrix metalloproteinase expression; (6) IGF signaling, which regulates cell proliferation, differentiation, apoptosis and play a role in tissue homeostasis [24] (Fig. 1). Radiotherapy induces changes in the CAFs secretome and influences the paracrine action of CAFs within the TME. Tommelein et al. found increased IGF signaling in radiation treated CAFs from CRC cancer. Insulin-like growth factor 1 (IGF-1) and Insulin-like Growth Factor Binding Proteins (IGFBP2) were found to be almost 3-fold higher in conditioned medium from irradiated CAF compared with the non-irradiated control [22]. The secretome of CAFs from human non-small cell lung carcinoma was changed after receiving ablative ionizing radiation (AIR, 1x18Gy) treatment. Angiogenic factors including angiopoietin, SDF-1 and thrombospondin-2 (TSP-2) were downregulated, while the expression of bFGF was upregulated. HGF, IL-6, IL-8, IL-1β, and TNF–α expressions were unaffected [25]. Hellevik et al. showed that ablative dose radiation (1 × 18 Gy) promoted MMP-3 and inhibited MMP-1 expression in CAFs, while the expression of other major MMPs were not affected [23] (Fig. 1).

### Radiation regulates the pro-tumorigenic capability of CAFs

Radiotherapy could influence the tumor promoting capability of fibroblasts in the tumor microenvironment. Grinde et al. investigated the pro-tumorgenic effect of irradiated CAFs in a murine xenograft model of lung cancer. Mice were injected with A549 lung tumor cells mixed with irradiated or control CAFs. Experimental groups were comprised of A549 cells alone, A549 co-injected with non-irradiated CAFs, A549 co-injected with single-high dose irradiated CAFs (1 × 18 Gy) and A549 co-injected with fractionated-irradiated CAFs (3× 6 Gy). The tumor promoting ability was abrogated in both groups with irradiated (3× 6 Gy or 1 × 18 Gy) CAFs (from NSCLC), suggesting that high dose fractionated RT (3× 6 Gy) or single ablative dose RT (1 × 18 Gy) may change the function of CAFs and impair their tumor promoting characteristics [26]. What about the effects of repeated low dose radiation on the tumor promoting abilities of CAFs? Unfortunately, the only studies documented were based on normal fibroblasts cultured from lung and breast tissues. Normal breast fibroblasts were irradiated at 5 cGy every 12 h to a cumulative dose of 10 Gy [13]. Normal lung fibroblasts were irradiated at 4 Gy up to a cumulative dose of about 50 Gy [27]. Repeated low dose radiation treatment to these cells induced a senescence-like phenotype which significantly promoted the growth of cancer cells in murine xenograft models [13, 27]. These studies were conducted before the concept of cancer-associated fibroblasts was widely accepted, so the conclusion could not be generalized to CAFs arbitrarily.

**Fig. 2:**
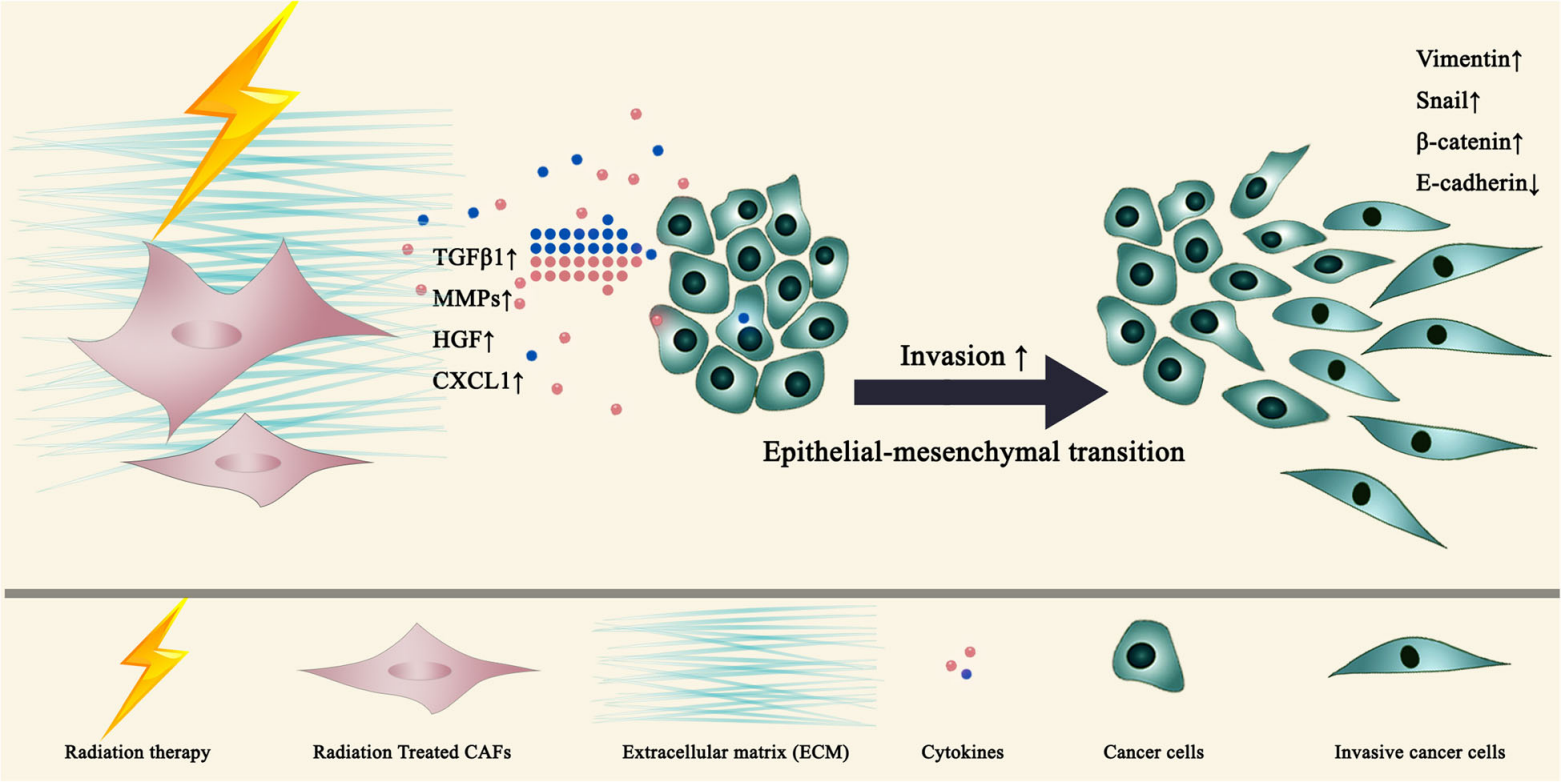
Enhanced expression of CXCL12, HGF, MMPs and TGF-β in irradiated fibroblasts induce invasiveness and epithelial-to-mesenchymal transition of cancer cells

Other studies suggest that irradiated fibroblasts may promote invasiveness and induce epithelial-to-mesenchymal transition in cancer cells (Fig. 2). TGF-β signaling in the tumor has been found to be elevated after radiotherapy and increased levels of circulating TGF-β post-radiation was likely a result of fibroblast activation. The upregulated TGF-β signaling subsequently stimulated the activation of the TME and the progression of tumors [4]. High levels of TGF-β1 have been detected in conditioned media obtained from irradiated fibroblasts, which promoted epithelial-to-mesenchymal transition in tumor cells indicated by increased expression of vimentin, snail and beta-catenin, as well as decreased E-cadherin expression [28]. Irradiated NIH 3 T3 fibroblasts release high levels of TGF-β1, which promote T3M-1 tumor cell invasion. In a collagen gel invasion assay, conditioned medium from fibroblasts receiving a single dose radiation of 1, 6 or 12Gy promoted T3M-1 cell invasion in a dose dependent manner with maximum effect achieved at 12Gy. Interestingly, conditioned medium from fibroblasts receiving 24 Gy single dose radiation failed to exert a more pro-invasive effect [29]. Using a matrigel invasion transwell assay, Kenoki Ohuchida et al. found that irradiated CAFs increased the invasiveness of pancreatic cancer cells in a radiation (single 0, 5 10 Gy) dose-dependent manner. This phenomenon was further validated in an orthotopic murine xenograft model of pancreatic cancer. Interestingly, no changes in the level of HGF was found in the CAF conditioned medium. Instead, the increased invasiveness may be a result of upregulated c-Met expression in the cancer cells which lead to increased MAPK activity. [30]. In another study, a single dose 4Gy radiation on CAFs induced EMT and promoted invasion in pancreatic cancer cells. Radiation exposure increased CAFs-derived CXCL12, a ligand of the C-X-C Motif Chemokine Receptor 4(CXCR4) in pancreatic cancer cells [31]. To explore the effects of irradiated fibroblasts on invasion of esophageal squamous cell carcinoma (ESCC) cells, CAFs/NFs were both treated with single 4Gy/8Gy radiation. Results indicated that conditioned medium from fibroblasts after irradiation enhanced the invasiveness of ESCC cells in a dose-dependent manner. Moreover, cancer cells treated with conditioned medium from irradiated CAFs (at 4 and 8 Gy) compared with NFs showed significantly larger number of invading cells [32].

### The impact of CAFs on radiotherapy: histopathological evidences

**Fig. 3:**
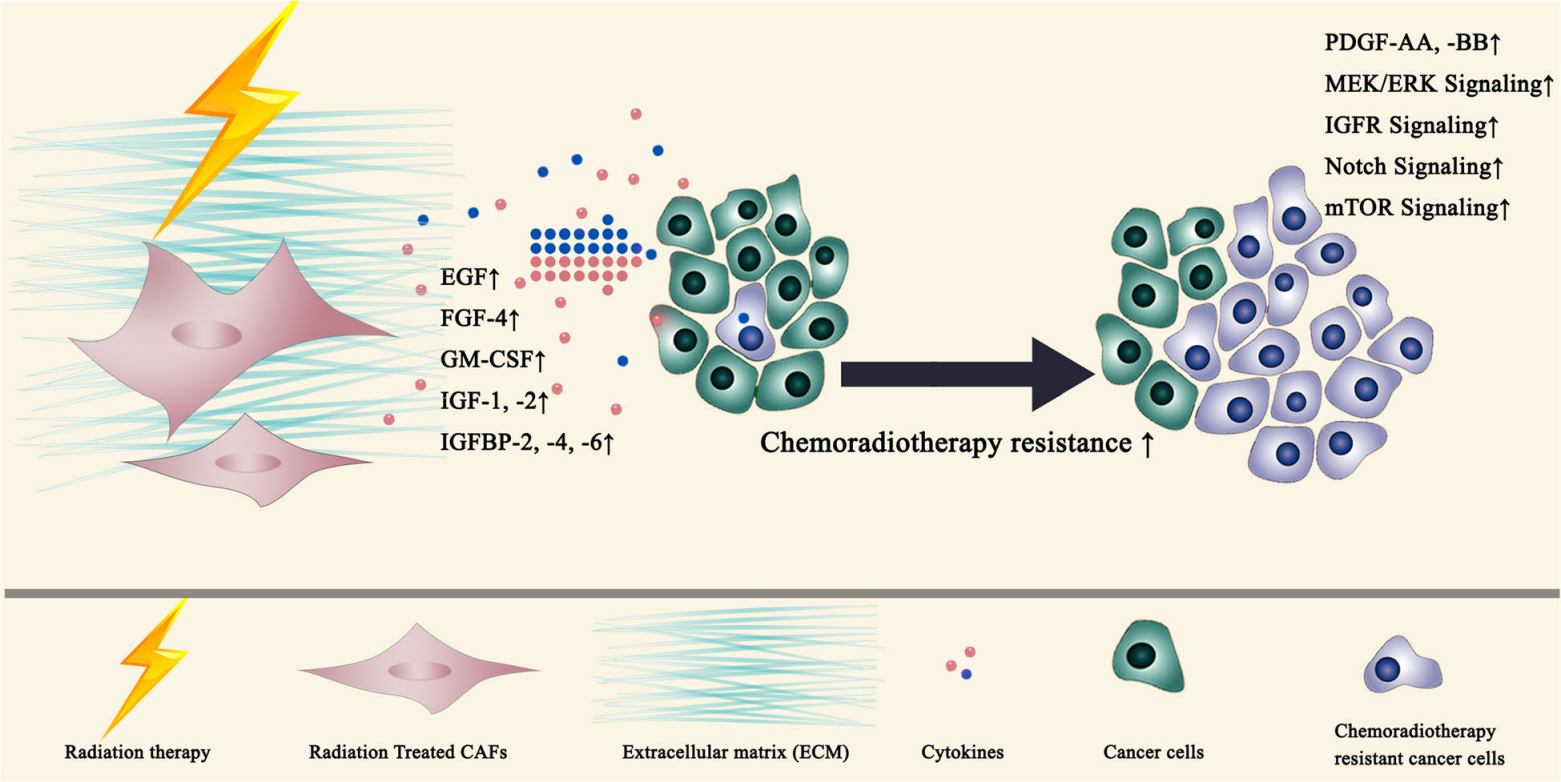
Enhanced expression of EGF, FGF-4, GM-CSF, IGF-1,2, IGFBP-2,4,6 in irradiated fibroblasts induce chemoradioresistance in cancer cells

Matsuoka et al. explored the relationship between the proportion of CAF component and tumor chemoradiotherapy response. Paraffin fixed tumor tissue sections from 60 patients with oral squamous cell carcinoma receiving preoperative 5-Fu based chemoradiotherapy were immune stained with CAF markers to identify the CAFs component within the tumor. High CAFs component was found to be correlated with advanced pT and pN stage (pathological TNM stage system) and poor prognosis compared to low CAFs component group [33]. The impact of CAFs on neoadjuvant chemoradiotherapy response in rectal cancer was investigated in several studies. α-SMA/neoplastic epithelial area ratio, FAP and SDF-1 were used as indicators for CAFs within the tumor. A larger CAFs population in the postoperative residual tumor tissue was found to be associated with poor recurrence-free survival [34, 35]. In another study, immunohistology staining of sections from 55 rectal cancer patients showed that mTOR, an IGF1-R signaling intermediate, was increasingly activated in patients after receiving chemoradiotherapy. Interestingly, high mTOR activation correlated with a high proportion of stromal CAFs, suggesting that chemoradiotherapy may indirectly elicit pro-survival signals through the stroma [22] (Fig. 3).

### CAFs promote radiotherapy resistance in vitro and in vivo

CAFs have been shown to confer radiotherapy resistance to cancer cells primarily through paracrine actions of secreted growth factors. Conditioned media from cervical squamous cell carcinoma derived CAFs increased the proliferation and clonogenic survival of irradiated HeLa cells. The protective effects were enhanced by using conditioned medium from mixed culture of CAFs and HeLa cells. Growth Factor Profiling of conditioned medium showed that the CAFs secreted IGF2, insulin-like growth factor binding proteins (IGFBP)-2, − 4 and − 6 and PDGF-AA which may confer a survival signal to overcome radiation-induced cell death [36]. CAFs have been shown to induce radioresistance of ESCC cells by overexpressing CXCL1. The secreted CXCL1 enhanced DNA damage repair of tumor cells by inhibiting ROS-scavenging enzyme superoxide dismutase 1 (SOD1) expression. Simultaneously, CXCL1 activates the prosurvival Mek/Erk pathway in the tumor cells. Such radioprotective effects were significantly reversed by using CXCL1 inhibitors [37]. Wang et al. showed that CAFs derived IGF1/2, CXCL12 and β-hydroxybutyrate significantly accelerated and enhanced the re-growth of irradiated lung cancer and melanoma cells in vitro and in mice xenograft tumor models by inducing autophagy. These factors increased the expression of reactive oxygen species (ROS) in cancer cells which stimulated PP2A activity and inhibited mTOR activation as a feedback. CAFs induced radiotherapy resistance in mice xenograft models was abolished using IGF2 neutralizing antibody or the autophagy inhibitor 3-MA [38]. In an attempt to identify genes associated with radioresistance, Nikolai N. Khodarev et al. selected a radioresistant head and neck cancer cell line SCC-61 by 8 continuous passages of xenograft implants in mice which was treatet with radiation. DNA array analysis of the resulting resistant strain identified an IFN-related DNA damage resistance gene signature (IRDS) in which STAT1 was demonstrated to be a key mediator of radioresistance [39]. A following study found that the IRDS was also associated with chemotherapy resistance in a panel of 34 different cancer cell lines and predicts recurrence of breast cancer after radiotherapy [40]. Further research found that stromal fibroblasts derived exosomes containing 5′-triphosphate RNA induce IRDS by activating RIG-1. Subsequent activation of STAT1 facilitated Notch signaling in breast cancer cells inducing a radiotherapy resistant stemcell-like phenotype. Increased STAT1/Notch signaling predicted clinical resistance to chemotherapy and radiotherapy in breast cancer [41]. Data from Tommelein et al. indicated that paracrine IGF-1/IGF-1 receptor (IGF-1R) signaling initiated by radiotherapy induced activation of CAFs, which enhanced CRC progression. Radiotherapy activated CAFs (10 fractions of 1.8Gy) promoted radiation treated (single dose 1.8Gy) COLO320DM colorectal cancer cell survival due to IGF-1R activation. During the CAFs mediated radiation protective process, a metabolic switch favoring glutamine consumption was detected in COLO320DM colorectal cancer cells. Results were confirmed in a CRC orthotropic model showing that the IGF-1R neutralization antibody sensitized radiotherapy by reducing organ metastases in mice [22] (Fig. 3).

### Targeting CAFs: a new perspective of treatment

The combination of targeting CAFs and radiotherapy has not been investigated to date. Since CAFs are naturally resistant while most tumor cells are sensitive to radiotherapy. The combination of targeting both populations may hold a promising future to radiochemotherapy. Current strategies for targeting CAFs include directly targeting and eliminating CAFs through antibody dependent recognition of specific cell surface markers, normalization of activated CAFs, targeting CAFs paracrine signaling pathways and targeting CAF-derived extracellular matrix proteins. Here, we briefly describe each of these strategies.

#### Directly targeting CAFs by cell surface marker

One approach for the elimination of CAFs is through antibody targeted therapy, which requires specific surface membrane markers. As mentioned earlier, a variety of membrane proteins such as FAP, and S100A4 are upregulated in CAFs in comparison to normal fibroblasts, these proteins may serve as potential targets. Even though an individual marker may not encompass the whole CAF population, partial elimination may have the potential to hinder tumor growth or abolish CAF associated therapeutic resistance.

FAP is a CAF expressed surface protein which has been studied extensively as a therapeutic target for CAF elimination. A humanized version of a murine mAb against FAP, F19 (sibrotuzumab; also known as BIBH 1) was clinically safe but showed limited activity in a phase II clinical trail of metastatic colorectal cancer. By conjugating the scFV chain of an FAP antibody to a photosensitive ferritin nanocage, the specificity of targeting FAP positive CAFs can be further enhanced [42]. As an extension of monoclonal antibodies, chimeric antigen receptor T cells (CAR-T), have also been used for the elimination of FAP positive CAFs. In a murine model of mesothelioma and lung cancer, infusion of FAP-CART resulted in growth reduction of the murine tumors in an FAP dependent manner. In a murine model of pancreatic cancer, FAP-CART reduced extracellular matrix proteins and glycosaminoglycans, decreased tumor vascular density and restrained tumor growth [43]. No FAP CAR-T clinical trials have been reported. However, the CAR-T industry has flourished with its successful treatment in leukemia. A variety of novel CAR constructs are under development to promoted better bioactivity of CAR-Ts. It is conceivable that these advancements may provide a more ready-to-use option for the targeting of FAP positive CAFs in future studies [44, 45].

S1004A is another CAF marker with potential therapeutic value. S100 belongs to one of the largest subfamilies of EF-hand calcium binding proteins, and S100A4 overexpression is strongly associated with tumor aggressiveness. S1004A is less specific than FAP as a marker for CAFs, it is expressed in macrophages as well as a fraction of epithelial cells. Monocloncal antibodies that neutralize S1004A function have been shown to reduces tumor growth and metastasis in a murine model of spontaneous breast cancer by inhibiting tumor initiation, premetastatic niche formation and by tuning the immune regulating balance between Th1 and Th2 cells [46]. In addition, an anti-S1004A antibody abolished endothelial cell migration, tumor growth and angiogenesis in immunodeficient mouse xenograft models of MiaPACA-2 and M21-S100A4 cells [47].

Meanwhile, novel CAF markers are being discovered. Tumor endothelial marker 8 (TEM8, also known as ANTXR1) was found to be highly expressed in cancer-associated fibroblasts and other stromal cells in a variety of cancers [48]. An ADC targeting TEM8 (m825-MMAE) blocks orthotopic pancreatic tumor growth as well as established colon and breast cancer metastases. Interestingly, the antitumor effect was conducted through an unexpected mechanism termed DAaRTS (drug activation and release through stroma), in which the prodrug was uptaken and accumulated in the stroma and released to exert its cytotoxic activity on adjacent tumor cells [48]. Other potential CAF surface markers include Podoplanin (PDPN) [49], PDGFRβ [50], TEM-1 [51], which are membrane proteins that require further characterization in tissue specificity before they can be used as reliable CAF markers.

#### Normalization ofactivated CAFs

As CAFs can generally be described as a group of constantly active fibroblast-like cells that support tumor progression (or restrain tumors in some cases), another approach to counter its biological function is by using agents that can reverted CAFs into a quiescent state or tumor-suppressive state. In patients with pancreatic ductal adenocarcinoma (PDAC), the deficiency of fat-soluble vitamins such as vitamin A and vitamin D are commonly seen. In a mouse model of PDAC, the administration of pleiotropic agent all-trans retinoic acid (ATRA) restored the retinol levels and repressed tumor growth. CAFs isolated from these tumors were reverted into an inactivated state. The inactivated CAFs inhibited tumor growth by suppressing tumor WNT–β-catenin signalling or increasing infiltration of CD8+ T cells [52, 53]. Interestingly, normalization of CAFs cound also be achieved by administration of vitamin D receptor ligand calcipotriol, which inhibited tumor progression in the PDAC models [54]. Calcipotriol inhibited stromal inflammation and fibrosis in the PDAC tumorsand enhanced gemicitabine delivery into tumor cells [54].

#### Targeting ***CAFs paracrine signaling pathways***

CAFs secrete a number of cytokines and growth factors to support tumor growth and regulate the malignant behavior of tumor cells. Some of these secreted proteins such as HGF and IL-6 have been studied extensively as therapeutic targets. HGF are stromal cell derived growth factors. Neutralization of HGF inhibited cancer cell invasion induced by stromal fibroblasts, suggesting a crucial role of HGF/Met signaling in the interaction between fibroblasts and tumor cells [11]. Both monoclonal antibodies and small molecular drugs targeting HGF/Met signaling have reached clinical trials. However, the results were mostly disappointing, suggesting that alternative treatment combinations may be required to exploit the full potential of targeting this pathway [55–57]. IL-6 is an inflammatory cytokine produced by cancer associated fibroblasts and other components of cancer microenvironment [58–60]. The IL-6/IL-6R/JAK/STAT3 pathway has been implicated in the development of breast cancer [61], colorectal cancer [62], lung cancer (NSCLC) [63], pancreatic cancer [64] and skin cancers [65]. Tocilizumab is a humanized monoclonal antibody targeting IL-6R. In preclinical studies, Tocilizumab exhibited anti-tumor activity towards ovarian, pancreatic and colitis associated colon cancer. A phase I clinical trail has demonstrated that the combination of Tocilizumab, interferon α2b and carboplatin or doxorubicin is safe and potentially effective for the treatment of ovarian cancer [66].

#### Targeting CAF-derived extracellular matrix proteins

CAF-mediated ECM remodelling causes desmoplastic reactions of tumor stoma which may promote tumor growth. Such desmoplastic tumor stroma could form a physical barrier and prevent the delivery of therapeutic agents into tumor cells, (e.g., PDAC [67]). Tenascin C is a ECM protein which modulates adhesion and pro-metastatic activity [68]. A tenascin C targeting agent, ^131^I-m81C6, which is an iodine-131 labelled murine mAb has been tested in phase II trials for recurrent malignant glioma patients. Compared to conventional treatment, ^131^I-m81C6 treatment following chemotherapy shows a survival benefit [69]. MMPs play an important role in CAFs mediated ECM remodelling [70]. More than 50 MMP inhibitors have been developed over the last decade. However, none of them succeeded in phase III clinical trials for anti-cancer treatment. Over 20 types of MMPs have been identified, and intensive research is required to figure out the role of each MMP family member in different tumors at different stages to guide the development of efficient MMPs inhibitors in future studies. Novel MMPs inhibitors have been produced and tested by early clinical trials [71].

## Future direction

The profound effects of radiotherapy on fibroblasts and cross talks between fibroblasts and the TME during radiotherapy remain largely unexplored. There are certain limitations in the studies mentioned in the review that need to be improve. Firstly, effects of radiotherapy on CAFs vary among different studies, which may be due to different radiation regimens of doses and fractions that were used. Most of the aforementioned studies used a few days or weeks to explore the short-term effects of radiotherapy on CAFs and tumor cells, whereas radiotherapy has its prolonged effects on tissue, even after several years. A comparative study exploring both the short term and long term effects of radiotherapy on tumor stroma in vitro and in vivo should be conducted in the future for further clarification. Secondly, CAFs are a group of heterogeneous cells of different origins and functions. Heterogeneous functions of CAFs in tumor progression include tumor supportive and tumor suppressive actions depending on their organ of origin, tumor type and disease stage. Although the tumor-suppressive mechanisms remain less clear than the tumor-promoting mechanisms [5, 10]. Studies directly comparing the response of paired normal fibroblasts and CAFs as well as different CAFs subtypes to ionizing radiation are urgently needed. Thirdly, Studies that focus on CAFs mainly used primary cultured cells from tumor specimens. These in vitro models cannot fully represent the physiological microenvironment where immune cells and primary tumor cells interact with CAFs through physical contact or paracrine actions. The in vivo animal models commonly used are co-injected with tumor cells and fibroblasts percutaneously or orthotropically. However, human fibroblasts are rapidly replaced by fibroblasts that originate from the recipients’ TME. Ideal models that evaluate radiotherapeutic effects on fibroblasts are still lacking. Transgenic mice that are ablated of CAFs (eg. S100A4+ cells depletion mice model [68]) seem to be valuable tools for exploring the permissive “soil” effect of CAFs on radiotherapy. Finally, there are numerous agents to target the biological function of CAFs in the lab and some them were assessed by clinical trials. The combination of targeting both populations may hold a promising future to radiochemotherapy.

## Conclusion

In summary, the above studies provide good preliminary data to illustrate the biological roles of CAFs in cancer radiotherapy. We support the notion that targeting CAFs as a supplementary treatment to conventional radiotherapy may improve treatment outcomes significantly. Further studies to validate this strategy in more physiological models may be required before continuing to clinical trial.

## Abbreviations

5-FU: 5-fluorouracil
AIR: Ablative doses of ionizing radiation
CAFs: Cancer-associated fibroblasts
CM: Conditioned media
CRC: Colorectal cancer
CRT: Chemoradiotherapy
EMT: Epithelial-Mesenchymal Transition
ESCC: Esophageal squamous cell carcinoma
Gy: Gray
HDGF: Hepatoma-derived growth factor
MAPK: Mitogen-activated protein kinase
NSCLC: Non-small cell lung carcinoma
OSCC: Oral squamous cell carcinoma
RC: Rectal cancer
RT: Radiotherapy
